# Vanishing Happiness: How Does Pollution Information Disclosure Affect Life Satisfaction?

**DOI:** 10.3390/ijerph19159530

**Published:** 2022-08-03

**Authors:** Penghu Zhu, Boqiang Lin

**Affiliations:** School of Management, China Institute for Studies in Energy Policy, Xiamen University, Xiamen 361005, China; penghuzhu@163.com

**Keywords:** pollution information, life satisfaction, cognitive effect, avoidance effect, envy effect, difference-in-difference

## Abstract

The role of information in energy and environmental policies is receiving extensive attention. This paper examines the impact of pollution information on residents’ life satisfaction and its channels in China. A difference-in-difference (DID) approach is used to match China Family Panel Studies (CFPS) data with information disclosure data to find the negative impact of PM_2.5_ information disclosure on residents’ life satisfaction. Heterogeneity analysis shows that the negative effects of information disclosure are more pronounced among young and middle-aged groups, residents with good physical conditions, higher education, higher income, and urban residents. The mechanism analysis indicates that the perception, avoidance, and envy effect are three important channels of influence. These findings provide some insights for public policy formulation aimed at enhancing the well-being of an entire population, such as paying attention to the psychological impact of policy implementation on different groups of people.

## 1. Introduction

For a long time, most countries have adopted an economic-developed strategy without controlling the pollution. The consumption of fossil energy improves the economy, but it also makes the environmental quality gradually decline, forming the environmental Kuznets curve [1]. At present, most countries are still facing severe environmental problems such as air pollution and water pollution. Since people can perceive air pollution more efficiently, many previous studies have found adverse effects of air pollution on human physical and mental health and welfare [2,3,4]. There is also some literature examining the changes in people’s behavior and subjective well-being in response to pollution [5,6,7,8]. However, when discussing the effects of pollution on individual perceptions and behavioral decisions, most of the literature ignores the difference between people’s perceived air pollution levels and measured pollution levels [9]; i.e., it ignores the role of pollution information.

The impact of pollution on well-being may be physical health such as lung disease or psychological health such as depression. Previous studies have found that these two effects tend to be negative, thereby reducing people’s happiness [10,11,12,13,14]. As for pollution information, its impact on well-being and channels may not be intuitive, because people’s response to pollution information is unclear. It has been widely recognized that information affects people’s emotions and behaviors. As the type of information available to people, pollution information will also affect people’s psychological emotions. In other words, when people face the real pollution information, their emotional changes will lead to changes in happiness.

However, most previous studies did not distinguish the differential effects of perceived and measured air pollution, and they did not examine the impact of changes in subjective air pollution. Most of them assume that people can recognize the accurately measured air pollution even though they do not point out it. However, the rationality of this assumption should be questioned, because people’s perceived and measured air pollution levels are often inconsistent [15]; that is, people may underestimate or overestimate the quality of the environment around them. Moreover, the two of them have different effects on subjective well-being. For example, Mackerron and Mourato [16] found that once residents are aware of the harm of air pollution to human health and the ecosystem, regardless of the level of measured air pollution, perceived pollution will directly damage residents’ well-being. Therefore, we believe that the disclosure of measured pollution information will correct people’s cognitive bias, which will cause changes in people’s subjective air pollution levels, thus affecting their well-being. Therefore, this paper examines the changes in people’s well-being before and after the disclosure of pollution information, regardless of whether the measured pollution level has changed in this process.

When pollution information is either not collected or deliberately withheld by the government, it is hard to respond to pollution for individuals, which is more severe in most developing countries [15]. China is a typical country that has this peculiar problem, where rapid industrialization and urbanization have produced severe environmental pollution, with air pollution being a major concern. During the 2000s, the daily average concentration of delicate particulate matter (PM_2.5_) exceeded 50 ug/m^3^, which is five times above the World Health Organization guideline [15]. According to the “2015 *Environmental Status Bulletin*” released by the Ministry of Environmental Protection, only 73 (21.6%) out of 338 Chinese cities met the air quality standard [17]. With the rapid growth of the social economy, governments are more likely to pay more attention to air pollution as well as the increment of public awareness. Correspondingly, the people’s demand for a better environment has become stronger [18,19]. During the period of 2013 to 2014, China gradually carried out a real-time air quality monitoring and disclosure program throughout the country, resulting in rapid and sudden changes in residents’ access to pollution information [17].

Based on a quasi-natural experimental context on the gradual disclosure of PM_2.5_ information in China, this paper examines the impact of pollution information disclosure on residents’ life satisfaction. We make three main contributions to the literature. First, we contribute to the literature on information and residents’ subjective feelings. To the best of our knowledge, studies that examine the role of pollution information remain scant. Barwick et al. [15] first examined the effect of pollution information disclosure on avoidance behavior, health, and house prices and estimated the value of pollution information disclosure items. However, it does not focus on the effect of pollution information on personal feelings. Personal feelings measured by subjective well-being or life satisfaction are an essential part of formulating and improving environmental policies for people’s increasing the pursuit of better environmental quality.

Second, most of the previous literature applies the cross-sectional data to detect the causal effect of pollution information, leading to a serious endogeneity. In contrast, this paper identifies the causal effect of pollution information by using a quasi-natural experiment approach, which is considered to avoid similar drawbacks [20]. Based on China’s specific pollution information disclosure context, this paper combines CFPS microdata with macro pollution data and uses a DID model to examine the causal effects, effectively addressing the endogeneity in the identification design. Moreover, since the CFPS data are the most representative and have abundant respondents with rich individual characteristics in China, it can essentially solve the problem of omitted variables and significantly improve the validity of our empirical findings.

Finally, this paper contributes to understanding the channels for pollution information distinguished from pollution on residents. The effect of pollution on well-being mainly affects people’s mood, causes respiratory diseases, or indirectly affects well-being through daily behavior [3]. Different from the effects of pollution, pollution information affects residents’ well-being in three ways: the first is to enhance or correct people’s environmental perceptions, the second is to promote changes in people’s daily behaviors, while the third is to contrast with other environments and affect subjective feelings. We examine each of these three channels, which can serve as a guide to public policy formulation to improve the welfare of residents.

The upcoming sections are organized as follows. Section 2 reviews the literature on air pollution and information effects; Section 3 introduces the background of PM_2.5_ information disclosure in China; Section 4 presents the empirical strategy and data; Section 5 discusses the results, and Section 6 concludes.

## 2. Literature Review

### 2.1. Effect of Air Pollution

It has become a consensus that air pollution increases the incidence of certain diseases, reduces life expectancy, and increases mortality in a great deal of literature. Pregnant women and infants may be more vulnerable to air pollution. Many pieces of literature find that air pollution has a negative impact on the rate of high-risk pregnancies among pregnant women, fetal malformations, and infant mortality [2,21,22,23]. Imelda [24] further distinguishes between indoor and outdoor pollution and finds that clean fuel substitution policies in Indonesia reduce the probability of infant mortality by 16% to 34%. Tian et al. [25] also find that indoor air pollution caused by the use of solid fuels has seriously damaged the health status of residents. The adverse effects of air pollution on other adults have also been confirmed, with studies concluding that air pollution leads to increased mortality due to cardiopulmonary system diseases and reduces people’s life expectancy [11,14,26]. In addition to physical health, air pollution can also affect mental health and induce mental disorders such as insomnia and depression [12,13]. Chen et al. [3] summarize that air pollution affects mental health in three ways: (1) air pollution can cause oxidative stress in the brain, disrupting the role of brain cytokines in mediating brain functions, including emotional neuronal circuits; (2) physical diseases caused by air pollution can also affect individuals’ mental health; (3) air pollution may also indirectly affect individuals’ mental health by reducing labor productivity and outdoor activities.

The impairment of physical and mental health can lead to other consequences. Chen et al. [12] find that air pollution increases the absence rate among elementary school students. Sager [27] finds that short-term health problems such as cognitive impairment and cardiopulmonary disease caused by air pollution exposure reduce drivers’ ability to drive safely, thereby increasing the probability of traffic accidents. What is worse, air pollution may exacerbate the severity of COVID-19 syndrome [28]. Other literature measures the additional health care costs associated with air pollution, showing that a significant amount of this cost tends to impose a heavier health care burden on individuals or countries [29].

People also change their behavioral decisions to respond to air pollution. Some scholars measure residents’ willingness to pay for clean air based on changes in migration behavior, daily necessities, and health care expenditures and use this to measure the social costs of pollution [18,30,31]. Currie et al. [32] find that the operation of a toxic plant decreases house prices within 0.5 miles of the plant by about 11%, and this negative effect continues after the poisonous plant is shut down. Qin et al. [33] find that air pollution significantly raises the purchase price of houses for migrant residents, and they believe that this is because the utility improvement brought by house purchase offsets the utility loss brought by air pollution, thus improving their willingness to pay for house purchase. Ito and Zhang [8] point out that as people become more health-conscious and have more robust demand for better air quality, their willingness to pay for clean air increases as well.

### 2.2. Effect of Information

Since information is often asymmetric and incomplete, exploring the nudge effect of information is critical in economics. Much literature has found that information cues or feedback can reduce residential energy consumption [34,35,36]. In terms of pollution, however, there is relatively little literature examining the effects of pollution information compared to reviewing the effects of air pollution on individuals. Graff Zivin and Matthew [37] find that air quality alerts significantly reduce the frequency of people’s outdoor activities. Jacobsen [38] and Tu et al. [39] examine changes in environmental awareness following the screening of environmental-themed documentaries in the United States and China, respectively. Both find that the availability of air quality information increases people’s willingness to pay for clean air. Barwick et al. [15] find that the disclosure of air quality information increases residents’ environmental awareness, thus influencing their behavioral decisions, such as changing their residential address or even increasing their housing expenditures, adjusting their consumption expenditures and lifestyle habits to avoid pollution. They further found that the annual benefits of risk-compensating behavioral changes from the disclosure of pollution information in China exceeded RMB 122 billion. Shen and Sun [40] found that the clarity of pollution information increases health care expenditures in China. Jalan and Somanathan [41] showed that households that were informed that their drinking water might be contaminated increased their expenses on water purification in India. There is also literature that examines the positive effect of pollution information disclosure on regional house prices [32,42,43]. Given the unanticipated economic shutdown and significant air quality improvement in many Chinese cities caused by COVID-19, Kahn et al. [44] find an increasing effect of clean air on public environmental concerns. Wang et al. [45] find that mandatory pollution information disclosure forces the government to control pollution, thereby increasing people’s happiness.

By summarizing the above literature, we found only one study in the literature examining the effect of pollution information on happiness [45], although some of the literature has examined that of air pollution [46,47,48,49]. Well-being is an indicator of an individual’s subjective evaluation of the quality of life. In addition to absolute and relative income as the main influencing factors, other inequality factors such as opportunity inequality [50] and environmental inequality [51,52] also affect well-being. Moreover, the increasing availability of pollution information might stimulate people to initiatively evaluate their own living environment. When bad news comes out from the pollution information, such a change would exacerbate the disappointment and further worsen subjective well-being [52]. Therefore, pollution information, distinct from pollution itself, has different channels of influence on individual feelings: first, it can enhance or correct people’s environmental cognition; second, it may promote changes in people’s daily behaviors by increasing their environmental awareness; and third, it can contrast with others’ environment and thus influence subjective feelings. The following section will verify these channels through a rigorous empirical strategy.

## 3. Institutional Background

Environmental pollution in China has become increasingly severe since the reform and opening up in 1978 [53,54]. However, the types of pollutants at different stages of development are somewhat different, and the focus of the corresponding environmental regulation policies has also changed. In 1982, the first Chinese ambient air quality standard was established, *Atmospheric Environment Quality Standard*, which stipulates that the primary pollutants are total suspended particles, coarse particulate matter (PM_10_), sulfur dioxide (SO_2_), nitrogen oxides (NO_x_), carbon monoxide (CO), and ozone (O_3_). However, until the 21st century, the main threat to air quality was sulfur dioxide from coal burning. In response, the Chinese government introduced the two-control zone policy (TCZ) in 1998, which specifies the designation of acid rain and sulfur dioxide pollution control zones throughout the country.

Starting in the early 2000s, particulate matter (PM) became the primary pollutant [55,56]. Thereafter, the government regulatory measures shifted to city-based air pollution reduction [57,58,59], but these efforts proved ineffective due to local governments’ strong competitive incentives for economic growth and poor monitoring and enforcement by the central government. After 2008, the increasing frequency of hazy weather in China, especially in northern cities during winter, attracted much attention from social media and the general public. The culprit behind the haze began to point to PM_2.5_, which is a pollutant that has long existed in China at high levels but is not clearly recognized by residents [17].

Frequent smog pollution and public focus on environmental quality promoted the introduction of policies. In February 2012, China’s Ministry of Environmental Protection (MEP, now Ministry of Ecology and Environment) issued the *Ambient Air Quality Standards*, which set a schedule for monitoring and releasing PM_2.5_ data, formally incorporating PM_2.5_ into the ambient air quality evaluation system, requiring localities to complete the installation of monitoring equipment and releasing real-time monitoring data. After that, the MEP introduced a three-phase implementation plan for the new air quality standards. The first step was to install new pollutant monitors in critical regions such as Beijing, Tianjin, and Hebei, the Yangtze River Delta, and the Pearl River Delta, as well as in municipalities and provincial capitals, and to make pollution data public by the end of December 2012. The second step was to carry out environmental monitoring in 116 cities, including key cities and model cities for national environmental protection, and to make the data public by the end of October 2013. The third step was to monitor and release new indicators in all prefecture-level cities by November 2014. In the actual implementation process, however, the China National Environmental Monitoring Center made public the PM_2.5_ real-time monitoring data of all cities nationwide in four batches, namely on 1 January 2013 (76 cities), 1 October 2013 (38 cities), 1 January 2014 (97 cities) and 1 January 2015 (179 cities) (http://www.cnemc.cn/en/, accessed on 1 October 2021).

**Figure 1:**
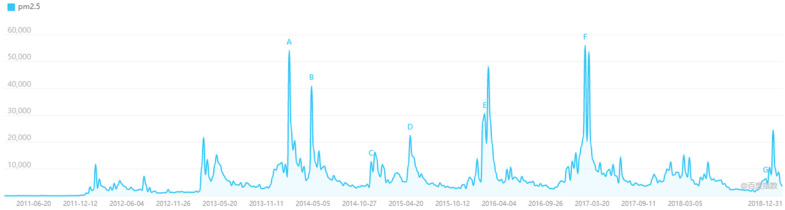
Baidu Index of PM_2.5_ from 2011 to 2018. (A–G) represent several concentration peaks of PM2.5 in recent years.

The real-time PM_2.5_ monitoring-and-disclosure program brings a flood of pollution information. With the media’s frequent broadcasting of PM_2.5_ data, the public’s cognition of PM_2.5_ experienced the process from scratch. Figure 1 shows the trend of search index for the term PM_2.5_ from 2011 to 2018 in Baidu, the largest search engine in China, which summarizes the number of queries for PM_2.5_ in China every day among both desktop and mobile users. It can be found that the first climb in search volume for the term PM_2.5_ occurred in December 2011, doubled at the end of 2012, and nearly tripled at the end of 2013 from its peak in 2012, with subsequent years also showing seasonal peak changes, which is consistent with the frequent occurrence of winter pollution in China. Figure 1 reveals the interaction between PM_2.5_ information disclosure and residents, which is likely to have a substantial impact on their daily life perceptions and behaviors. The following section examines the effects of information disclosure on residents’ life satisfaction using a quasi-natural experiment design.

## 4. Methodology

### 4.1. Identification Strategy

Following Barwick et al. [15], we examine the effect of pollution information disclosure on residents’ life satisfaction using a differences-in-differences method with Equation (1).

(1) $$Lifesati_{ijt}=\beta_{0}+\beta_{1}\cdot DID_{jt}\times PM_{2.5}{}_{jt}+\beta_{2}\cdot DID_{jt}+\beta_{3}\cdot PM_{2.5}{}_{jt}+\lambda \cdot X_{ijt}+\delta_{i}+\gamma_{t}+\varepsilon_{ijt}$$

where $Lifesati_{ijt}$ denotes the life satisfaction of person i in city j and year t. The dummy variable $DID_{jt}$ takes the value one for all periods after city j implements the information program. $PM_{2.5}{}_{jt}$ denotes the annual average PM_2.5_ concentration of each city taking the logarithm. Vector $X_{it}$ includes the set of individual, household, and city-level control variables, $\delta_{i}$ is the individual fixed effects, $\gamma_{t}$ is the year fixed effects, and the last term $\varepsilon_{it}$ denotes the remaining unexplained shocks. The main coefficient we pay attention to is $\beta_{1}$, which represents the change of the impact of pollution on well-being after the disclosure of pollution information. The economic implication is that the fraction of the population’s life satisfaction increases for every 1% increase in pollution levels after the information is made public. In particular, we are interested in the causal effect of the information disclosure ($\beta_{1}$), rather than the effect of PM_2.5_ per se ($\beta_{3}$). The OLS estimate of $\beta_{1}$ is consistent even though the possible endogeneity is caused by omitted variables or errors in the measurement of pollution exposure (the detailed proof process can be seen in Barwick et al. [15]).

### 4.2. Data and Variables

#### 4.2.1. CFPS

The micro dataset for this paper comes from the CFPS, which is a nationally representative longitudinal survey implemented by the Peking University every two years. Using the survey data collected at the individual, family, and community levels, many papers have studied various aspects of income, education, health, and energy using CFPS data [52,60,61,62]. We used unbalanced panel data from 2010 to 2018 to obtain about 158,760 observations by matching the individual and household pools. Among all observations, 17,261 spans all five surveys. The main variable, life satisfaction, is defined as follows.

Life satisfaction is usually defined as an individual’s subjective evaluation of their overall life situation. In the CFPS questionnaire, its question is “Are you satisfied with your life?” using a scale of 1 (very unsatisfied) to 5 (very satisfied) score. In our sample, most respondents score more than 3, with 32.91%, 30.40%, and 24.64% for 3, 4, and 5, respectively, and about 12% in total for individuals with scores below 3. Chinese residents’ average life satisfaction score is 3.64, which is consistent with Asadullah et al. [63] and Zhang et al. [52].

Note that household and individual characteristics are always the essential covariates in assessing the determinants of life satisfaction [52]. Following the relevant literature, this paper introduces gender, age, political identity, marital status, educational level, health status, work status, absolute income, relative income, household registration, household size, housing area, and the number of houses as the control variables. Descriptive statistics of these variables are presented in Table 1.

#### 4.2.2. PM_2.5_

To investigate the effect of information disclosure on life satisfaction with the DID model, the PM_2.5_ dataset before and after information disclosure should be obtained. Since China did not collect its own PM_2.5_ dataset before the exposure, we derived from the gridded annual average PM_2.5_ concentration from 2000 to 2016 at a spatial resolution of 0.01 degrees from the Atmospheric Composition Analysis Group (Atmospheric Composition Analysis Group. Surface PM_2.5_. (http://fizz.phys.dal.ca/~atmos/martin/?page_id=140#V4.CH.03, accessed on 1 July 2020)). The QGIS software is used to extract data from the global PM_2.5_ grid map combined with the administrative regions vector map in China, and the PM_2.5_ pollution data of each prefecture-level city are obtained.

However, gridded data for 2018 were unavailable. Hence, referring to Yue et al. [64], we extrapolated the gridded PM_2.5_ concentration in 2018 based on gridded data in 2016 and the ratio of monitoring data between 2016 and 2018. The monitoring PM_2.5_ concentration was derived from China’s National Urban Air Quality Real-Time Publishing Platform. Due to space limitations, details of the steps can be found in Yue et al. [64].

#### 4.2.3. PM_2.5_ Disclosure Data

As mentioned earlier, the actual PM_2.5_ data disclosure schedule is not in line with the Ministry of Environmental Protection’s plan, and hence, we had to collect it at a specific time for each city in the actual implementation. The real-time PM_2.5_ monitoring data for all cities in China were disclosed in four batches on the website of the China National Environmental Monitoring Center: 1 January 2013 (74 cities), 1 October 2013 (40 cities), 1 January 2014 (97 cities) and 1 January 2015 (179 cities). The CFPS sample contains residents of 128 cities, and the number of cities disclosing PM_2.5_ data at the four time points was 36, 12, 19, and 48, respectively. When a city discloses PM_2.5_ information, all residents of this city are able to access pollution information.

Since the CFPS was conducted every two years where each survey basically starts in April and ends in August of the same year, we match the first and second batch to cities with the CFPS data in 2014 considering the information lag effect. We find that a total of 12,926 residents obtained pollution information in the data in 2014. We then matched the third and fourth batch of cities with the CFPS data in 2016 and observed that 19,694 new residents obtained the pollution information. The following parts also test the robustness of the processing effect without considering the lag effect of the information.

#### 4.2.4. Other Urban Characteristics

In addition to the individual and family characteristics, the exclusion of some characteristics at the urban level was likely to threaten the robustness of our empirical specification, and therefore, we controlled for differences in economic levels in different regions. Following Shen and Sun [40], we obtained four urban level control variables from China City Yearbooks: per capita GDP, per capita wage, population density, and per capita green space area. The definition and descriptive statistics for each variable are shown in Table 1.

## 5. Results and Discussions

### 5.1. Main Results

Table 2 presents the baseline regression results of Equation (1). From Column (2) to Column (4), we add characteristic variables at the individual, family, and city level in sequence based on Column (1); in Column (5), we cluster standard errors at the individual level to allow for arbitrary serial correlations among the sample periods; and in Column (6), we further relax the assumption of random disturbance items with clustering standard errors to the family level. All specifications control for individual and time-fixed effects.

It can be found that the negative effect of pollution information disclosure on residents’ life satisfaction is significant, regardless of increase in control variables or changes in clustering level of standard errors. We focus on Columns (4)–(6), from which we find that for every 1% increase in PM_2.5_ annual average concentration, information disclosure reduces life satisfaction by 0.04 points, or about 1.21% (0.04 divided by the average life satisfaction of all residents 3.64). This is consistent with the findings of Zhang et al. [52], who found that Internet use reduces happiness because of the decline in satisfaction with environmental quality after obtaining pollution information. However, this is contrary to the conclusion of Wang et al. [45], who found that mandatory air quality information disclosure improved people’s well-being. They believe that this is due to the government’s strengthened supervision on pollution control of enterprises, thereby reducing air pollution. The reason why their conclusions differ from ours is that they used data before 2013, when China’s Internet was not widely popularized, and people could not obtain timely pollution information. When they obtain the information, the air quality may have been improved and the achievements of environmental governance have been disclosed. Therefore, this will lead to an increase in people’s happiness. This article is an important supplement to this literature. We believe that the decline of people’s happiness after obtaining real-time information of pollution information is caused by environmental awareness and people’s response to it. From this point of view, this is consistent with the conclusion of Barwick et al. [15]. The following will further test our hypothesis.

### 5.2. Validity and Robustness Checks

#### 5.2.1. Parallel Trend Test

**Figure A1:**
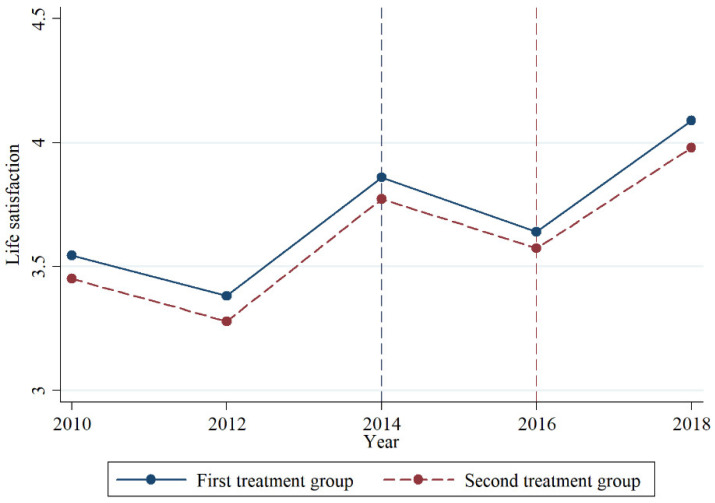
Changes in life satisfaction of different groups.

The DID model requires that the samples of the treatment group and control groups’ samples have similar trends before the pollution information disclosure. We first compare the trend in life satisfaction of residents in the first batch and the second batch of treatment groups from 2010 to 2018 (Figure A1 in the Appendix A). We find that before 2014, the two groups’ life satisfaction trends were approximately consistent, but they were no longer parallel after 2014, indicating that the disclosure of pollution information works. To further verify whether the two sets of samples met the parallel trend hypothesis, we adopted the event study method to replace the DID variable in Equation (1) with a series of period dummy variables in Equation (2).

(2) $$Lifesati_{ijt}=\alpha_{0}+\sum_{k=-3,k\neq 0}^{k=2}\beta_{k}\cdot DID_{jt}^{k}\times PM_{2.5}{}_{jt}+\alpha_{1}\cdot PM_{2.5}{}_{jt}+\lambda \cdot X_{ijt}+\gamma_{t}+\delta_{i}+\varepsilon_{ijt}$$

where $DID_{jt}^{k}$ indicates whether the city j in the year of t is in the k period before and after the pollution information disclosure. It needs to be pointed out that the event study method usually chooses the previous period of policy implementation as the benchmark to eliminate the interference of the current period. However, the survey date of CFPS in this paper lagged behind the pollution information disclosure date, so we used the current pollution information disclosure period as the benchmark. The definitions of other variables in Equation (2) are the same as those in Equation (1).

**Figure 2:**
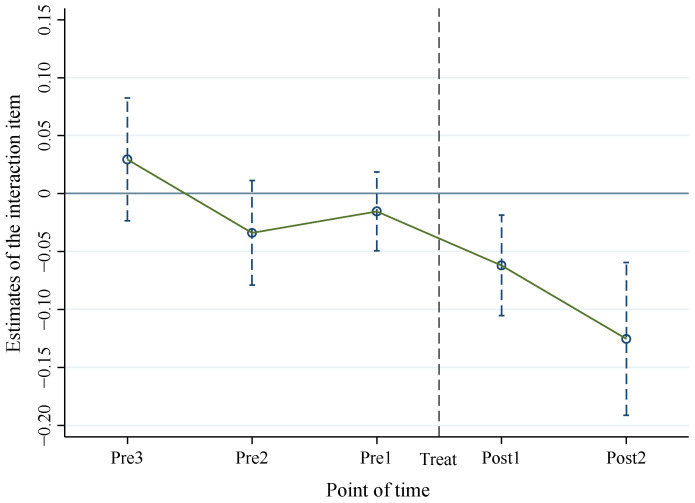
Changes in life satisfaction in pollution information access.

The regression results are shown in Figure 2 (the specific coefficients are shown in Table A1). It can be found that before the pollution information is disclosed, the time dummy variables were not significant (Pre3, Pre2, Pre1), and that the first period (Post1) and second period (Post2) after the disclosure were significantly negative. This shows no significant difference in the trend of life satisfaction between the treatment group and the control group before treatment, which meets the preconditions of the DID model.

#### 5.2.2. Placebo Test

**Figure 3:**
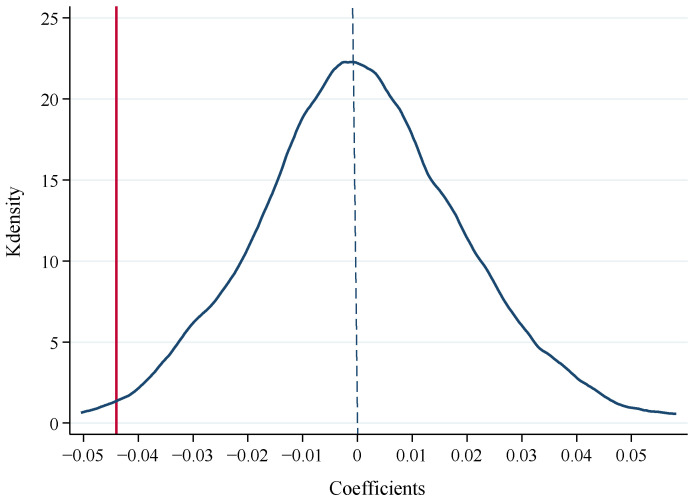
Kernel density distribution of treatment effect for 500 times.

We conducted a placebo test to strengthen the reliability of the above results. We randomly set different information disclosure time for the residents of each city and then repeated the estimation for Equation (1) 500 times. Figure 3 shows the kernel density curve of $\beta_{1}$ obtained from 500 regressions, and the vertical solid red line represents the actual effect estimation value in the preceding paragraph. It can be found that the coefficients are approximately normally distributed around 0, which is significantly different from the actual effect. This indicates that our estimation result is unlikely to be obtained by accident. That is, it is unlikely to be affected by other policies or random factors, and the negative effect of pollution information is real.

#### 5.2.3. Robustness Checks

As shown in Table 3, we change the treatment group, eliminate the possible interference samples, and adopt different models for estimation to conduct robust checks.

Cities that publish pollution information in different batches are not random, and their information disclosure methods are different. Therefore, we need to consider the possible differences in causal effect between treatment groups. In Column 1, we include the residents in the third batch of cities that disclosed pollution information as the treatment group in 2014. The results show that the coefficient of the interaction term is significantly negative at the level of 5%. The effect is smaller than the baseline result, indicating that the negative effect of information disclosure on residents’ life satisfaction is more significant if the information lag effect is considered. In Column 2, we retained the residents in the first batch of cities that disclosed pollution information as the treatment group in 2014 and set the fourth batch as the control group in 2016. The treatment effect was still significantly negative, and there was little difference from the result in Table 3.

Since residents living in municipalities and provincial capitals in China may have a higher quality of life, their response to environmental information may be more sensitive. Thus, we eliminated the residents from this part to re-estimate in Column 3 and found that the results were still not much different from the previous results, which indicates that the effect of pollution information disclosure on life satisfaction does not vary from city to city.

In Column 4, we further eliminate the interference of indoor pollution. People exposed to indoor pollution all year round may be less sensitive to outdoor pollution, which may cause our underestimation [65]. Referring to Jia et al. [59], we define residents who use solid fuel, firewood, and coal to cook as individuals who have polluted indoors. After removing this part of the sample, we repeated the regression. The results showed that pollution information disclosure’s negative effect was greater; i.e., for every 1% increase in the annual average concentration of PM_2.5_, residents’ life satisfaction drops by more than 0.05 points. This proves our conjecture that people who do not usually perceive pollution will suffer a relatively more significant effect from pollution information disclosure.

In Column 5, since our life satisfaction indicator is only 1–5 points, a total of five values, we use an ordered probit model to estimate Equation (1) based on the practice of Zhang et al. [52]. What needs to be pointed out is that the ordered probit model cannot control individual fixed effects. However, the regression results still reveal the negative effect of pollution information disclosure.

#### 5.2.4. Endogeneity Discussion

In addition to the above robust checks, we further discuss potential endogeneity. In this study, after the cities disclose pollution data, the degree of urban pollution may be affected by unobserved factors related to PM_2.5_, such as the government’s environmental governance efforts. These missing variables may affect the happiness of residents and the pollution level simultaneously, which leads to biases in the estimation results.

We use the annual number of days with a thermal inversion as an instrument for pollution to deal with endogeneity [2,3]. Temperature inversion is usually determined by the geographic and seasonal features of the city, and it is not subject to human control. However, temperature inversion will make it difficult for pollutants to diffuse, leading to an aggravation of pollution. Thus, the inversion meets the exogenous and correlation requirements as an instrument for pollution.

The temperature inversion data come from NASA. Consistent with Chen et al. [18], we calculate the temperature difference between 320 m above the ground and 110 m above the ground observed every 6 h. If the value is positive, it is recorded as 1; otherwise, it is recorded as 0. Based on this, the total frequency of temperature inversions in various places in the year can be obtained. As shown in Table 4, Columns (1), (3), and (5) are the regression results when the total frequency, the total frequency of the logarithm, and the proportion of total frequency in the whole year are used as the independent variables of PM_2.5_, respectively. It can be found that the effect of temperature inversion on pollution is significantly positive. Columns (2), (4), and (6) are the IV estimation results corresponding to Columns (1), (3), and (5), respectively, from which we can find that information disclosure still has a significant negative impact on life satisfaction, indicating that the findings above are reliable.

### 5.3. Heterogeneity Analysis

The above treatment effects may depend on people’s sensitivity to pollution and their ability to avoid pollution. As shown in Table 5, we further analyze the heterogeneity among residents with different characteristics, including age, health status, education, and income, urban and rural areas. First of all, Columns (1) and (2) show that the negative impact of information disclosure only works among younger groups, which may be because young people are more concerned about environmental quality and pollution information, and they are more motivated to avoid air pollution. Columns (3) and (4) are residents of the less healthy and healthy groups, respectively (the health is divided into seven levels in the questionnaire, and we will regard the first five as the less healthy group and the latter two as the healthy group). In the healthy group, information disclosure has a negative impact. This may be because healthy residents pay more attention to potential health risks. Of course, since our data cannot distinguish the specific diseases of less healthy residents, this may have some particular bias. For example, people with respiratory diseases may worry more about their health after information disclosure since the harmful effects of air pollution on respiratory infections have been found in some studies.

Columns (5) and (6) are residents with different education levels with the primary school as the dividing line. It can be found that only residents with higher education levels will be negatively affected by information disclosure. This may be because these residents are more concerned about environmental quality and pollution information, and they are more willing to spend money to avoid pollution. Columns (7) and (8) are divided by the median monthly income. It can be found that only higher-income residents are negatively affected with larger regression coefficients. Higher-income groups are more likely to have higher requirements for environmental quality, and hence, information disclosure decreases their life satisfaction. This finding is basically consistent with Zhang et al. [52]. They believe that although high-income people have better living conditions, it is difficult for them to change their surrounding environment in the short term, thus reducing their life satisfaction. This is also consistent with the results by education level, because generally the higher the education level, the higher the income of residents. Columns (9) and (10) are the heterogeneous results of urban and rural residents, respectively. The results show that only urban residents will be affected by information disclosure. This may be because the living situation of rural residents in China is much lower than that of urban residents, thus placing less attention on air pollution information and its possible damage [52].

### 5.4. Mechanism Analysis

In this section, we want to know why the disclosure of pollution information will cause the decline of residents’ well-being. After all, the motivation for the authorities to disclose pollution information is to promote pollution management to ultimately improve people’s well-being ratio rather than damage it. Therefore, only by understanding the mechanism can we avoid misunderstanding of such public policies or reduce their unexpected negative effects.

As mentioned in Section 2.2, the effect of pollution information on residents’ life satisfaction is different from that of pollution itself. The first is the cognitive effect: when residents do not know the actual pollution status, they will have a greater probability of underestimating the pollution level [15]. The actual information resulted in a decline in life satisfaction evaluation thereafter. The second is avoidance effect: after obtaining pollution information, to avoid the health risks of pollution, people will pay a certain price to try to reduce the negative effect of pollution, such as reducing the frequency of going out or increasing such preventive investments as buying air purifiers, masks, and other avoidance behaviors [40], which may cause a decline in the subjective evaluation of the quality of life. The third is the envy effect: people can obtain the pollution data of other regions that are better than their own, which may cause an envy effect and further aggravate the negative impact of pollution information. We verify whether these three channels exist or not respectively (It should be noted that the avoidance and envy effect may be considered as the outcome of cognitive effect, which will cause bias in the estimation of cognitive effect. Because only after residents perceive the pollution information will they make avoidance behavior or envy others. However, the cognitive effect here refers to the psychological gap of residents who realize that their environment is worse than previously expected. In other words, this is not caused by comparison with others or other reasons but only by comparison with themselves. We believe that this part of cognitive effect is independent of avoidance and envy effect. Unfortunately, due to the limitations of our data, our selection of cognitive variables may not accurately measure cognitive effects, which may lead to mixing with other effects. We thank the reviewers for this useful suggestion).

We verify the changes in the residents’ subjective evaluation of environmental pollution before and after the pollution information disclosure for the cognitive effect. The CFPS asked the following questions, “In general, how seriously do you think environmental problems are in China?”. Responses were given on an 11-point scale ranging from 0 = “not serious” to 10 = “very serious”. If the residents feel that the air quality is poor after the information is disclosed, then their subjective evaluation of environmental pollution will deteriorate, which will lead to a decline in their evaluation of the quality of life. As shown in Columns (1)–(3) of Table 6, for every 1% increase in the annual average PM_2.5_ concentration, the residents’ subjective pollution evaluation increases by 0.25 points after the information disclosure, indicating that the cognitive effect is likely to exist. This is consistent with Zhang et al. [52], who found that people’s exposure to more information will reduce environmental satisfaction.

We refer to Shen and Sun [40] to examine the avoidance effect of information disclosure on health care expenditures, indirectly indicating residents’ defensive investment. Columns (4)–(6) of Table 6 are the estimated results, from which we can find that after the information disclosure, residents’ health expenditures increase significantly, and thus, the avoidance effect is verified. For every 1% increase in the average annual concentration of PM_2.5_, residents’ health expenditures increase by 28.39 yuan every year after the information is disclosed. This is consistent with Shen and Sun [40], who also found that pollution information disclosure increased health expenditure. These additional avoidance costs lead to a decrease in life satisfaction.

Finally, we test the envy effect. It is difficult for us to directly test this effect because there is no question to directly ask residents whether they feel that the air quality in their place is better or worse than elsewhere. However, if the envy effect exists, residents living in areas with higher air quality should not be negatively affected by pollution information disclosure, and even after information disclosure, their life satisfaction will be increased due to comparison with others, while people living in low-quality areas may be more negatively affected. We divide the actual pollution level into low, medium, and high levels according to the 25%, 25−75%, and 75% quantiles, and respectively estimate Equation (1). As shown in Table 7, residents’ life satisfaction with lower pollution levels improves after the pollution information is disclosed, while residents with medium and high pollution levels are all negatively affected by information disclosure, and those with higher pollution levels experience worse effects. This is in line with our expectations and indirectly verifies the envy effect.

## 6. Conclusions and Policy Implications

Based on the quasi-natural experimental approach, this paper investigates the effect of pollution information disclosure in China on residents’ life satisfaction and finds that it significantly reduces residents’ life satisfaction through the cognitive effect, avoidance effect, and envy effect. For every 1% increase in the average annual concentration of PM_2.5_, pollution information disclosure reduces residents’ life satisfaction by 0.04 points, accounting for about 1.21% of residents’ average life satisfaction. This effect is more pronounced among young and middle-aged groups, residents with good physical conditions, higher education, higher income, and urban residents. Many developing countries, including China, are facing severe environmental pollution, as the formulation of better environmental policies remains their primary focus. Thus, the above findings provide some insights into the public policy toward information.

Firstly, environmental policies aimed at improving residents’ welfare may bring unexpected negative effects early on. With the increase in people’s income, their demands for higher environmental quality become stronger and stronger, which obliges the government to strengthen environmental regulation and increase administrative transparency in environmental governance. It is expected that with the advancement of environmental management, the welfare of residents will gradually increase. However, before environmental information disclosure improves environmental quality, this may also lead to the decline in residents’ short-run subjective satisfaction, since they are aware of their living conditions early, i.e., in regions with poor environments. While strengthening environmental governance, the government can give the necessary policy publicity at the initial implementation stage of similar policies to enhance their confidence in environmental management.

Secondly, environmental policies need to consider the pursuits of different groups to avoid the decline in life satisfaction caused by pollution avoidance behavior. After residents obtain pollution information, engaging in avoidance behavior has a particular cost, which may increase the economic burden on low-income groups and reduce their life satisfaction. Furthermore, low-income groups are more likely to be exposed to pollution, such as cooking with solid fuel and working with more pollution exposure [59]. The government can consider providing pollution subsidies to low-income and high-pollution exposed groups, such as issuing free masks. More importantly, it is necessary to emphasize the benefits of avoiding behavior on long-term health to alleviate the temporary psychological discomfort of these residents after disclosing information.

Finally, the government should continue strengthening environmental regulations to reduce the long-run negative effect of information disclosure. As the negative impact of information disclosure increases with the aggravation of pollution, if residents cannot see the improvement of environmental quality from the disclosed information, their happiness may be further reduced. Therefore, the government should increase investment in environmental governance, respond to residents’ environmental demands, improve residents’ sense of participation in the governance process, and minimize the negative impact of environmental pollution. In addition, for residents’ temporary dissatisfaction with environmental quality, the authorities should give a detailed environmental governance plan to eliminate their doubts.

This paper also has some limitations indeed. There is a specific interval between the survey time of CFPS data and the disclosure time of pollution information, which may reduce residents’ sensitivity to pollution information, and then, the estimated results may be underestimated compared with the disclosure time. However, the substantial evidence in this paper still shows that the negative effect of pollution information is real and should be taken seriously in the formulation of relevant policies.

## Figures and Tables

**Table 1:** Descriptive statistics.

Variables	Definition	Mean	S.D.
**Personal Information**			
Life satisfaction	Five categories: 1 = very unsatisfied and 5 = very satisfied	3.64	1.06
Urban	Urban = 1, rural = 0	0.47	0.50
Gender	Male = 1, female = 0	0.49	0.50
Political identity	Party member = 1, else = 0	0.08	0.26
Age	Age of people over 16 years old	46.04	16.70
Education	Years of education	7.47	4.81
Retire	Retire = 1, else = 0	0.12	0.32
Health status	Seven categories: 1 = very unhealthy and 7 = very healthy	5.42	1.27
Marital status			
Unmarried	Unmarried = 1, else = 0	0.14	0.34
In marriage	In marriage = 1, else = 0	0.79	0.41
Divorce or widowed	Divorce or widowed = 1, else = 0	0.07	0.26
Relative income	Five categories: 1 = very low and 5 = very high	2.45	1.04
**Family Information**			
Family income	Month income of family (yuan)	4004.23	3394.33
House area	Housing area (m^2^)	132.77	91.19
Family size	Number of household population	4.28	1.95
Other properties	More than 1 property = 1, else = 0	0.18	0.38
**Environment Information**			
PM_2.5_	PM_2.5_ concentration (ug/m^3^)	43.75	18.93
Per GDP	Per capita GDP (yuan)	48,584.45	31,295.92
Per capita wage	Per capita wage (yuan)	52,691.74	21,904.66
Population density	Population density (10,000 people/km^2^)	0.06	0.06
Per capita green area	Per capita green area (hectare/10,000 people)	21.37	31.77

**Table 2:** Baseline results.

	Dependent Variable: Life Satisfaction					
	(1)	(2)	(3)	(4)	(5)	(6)
DID * PM_2.5_	−0.043 ***	−0.043 ***	−0.043 ***	−0.044 ***	−0.044 ***	−0.044 **
	(0.011)	(0.012)	(0.012)	(0.015)	(0.016)	(0.017)
DID	0.159 ***	0.165 ***	0.168 ***	0.170 ***	0.170 ***	0.170 **
	(0.045)	(0.047)	(0.048)	(0.060)	(0.063)	(0.068)
PM_2.5_	0.007	0.008	0.003	−0.043	−0.043	−0.043
	(0.025)	(0.026)	(0.026)	(0.032)	(0.034)	(0.037)
Urban		−0.022	−0.021	−0.019	−0.019	−0.019
		(0.016)	(0.017)	(0.018)	(0.017)	(0.019)
Gender		0.023	0.027	0.034	0.034	0.034
		(0.106)	(0.110)	(0.117)	(0.128)	(0.122)
Political identity		−0.000	−0.003	−0.012	−0.012	−0.012
		(0.026)	(0.027)	(0.028)	(0.027)	(0.027)
Age		−0.013 *	−0.012	−0.010	−0.010	−0.010
		(0.007)	(0.008)	(0.008)	(0.009)	(0.010)
Education		−0.012 ***	−0.013 ***	−0.015 ***	−0.015 ***	−0.015 ***
		(0.004)	(0.004)	(0.004)	(0.005)	(0.005)
Retire		0.019	0.025	0.018	0.018	0.018
		(0.033)	(0.034)	(0.035)	(0.033)	(0.034)
Health status		0.039 ***	0.038 ***	0.038 ***	0.038 ***	0.038 ***
		(0.003)	(0.003)	(0.003)	(0.003)	(0.003)
Relative income		0.193 ***	0.191 ***	0.190 ***	0.190 ***	0.190 ***
		(0.003)	(0.003)	(0.003)	(0.004)	(0.004)
Marital status (taking “unmarried” as reference)						
In marriage		0.006	0.010	0.011	0.011	0.011
		(0.021)	(0.022)	(0.023)	(0.023)	(0.023)
Divorce or widowed		−0.077 **	−0.059 *	−0.068 **	−0.068 *	−0.068 *
		(0.030)	(0.031)	(0.033)	(0.035)	(0.035)
Family income			0.021 ***	0.020 ***	0.020 ***	0.020 ***
			(0.004)	(0.004)	(0.004)	(0.004)
House area			0.029 ***	0.026 ***	0.026 ***	0.026 **
			(0.009)	(0.009)	(0.009)	(0.010)
Family size			0.021 *	0.032 ***	0.032 ***	0.032 **
			(0.011)	(0.012)	(0.012)	(0.013)
Other properties			0.027 ***	0.024 **	0.024 ***	0.024 **
			(0.009)	(0.009)	(0.009)	(0.010)
Per GDP				−0.023	−0.023	−0.023
				(0.030)	(0.032)	(0.035)
Population density				0.052	0.052	0.052
				(0.059)	(0.055)	(0.058)
Per capita green area				−0.021 *	−0.021 *	−0.021
				(0.012)	(0.012)	(0.013)
Per capita wage				−0.110 **	−0.110 **	−0.110 **
				(0.050)	(0.051)	(0.056)
Constant	3.450 ***	3.461 ***	3.109 ***	4.812 ***	4.812 ***	4.812 ***
	(0.094)	(0.325)	(0.352)	(0.697)	(0.731)	(0.791)
Year	Yes	Yes	Yes	Yes	Yes	Yes
Individual	Yes	Yes	Yes	Yes	Yes	Yes
*N*	158,760	136,022	130,234	117,086	117,086	117,086

Note: Standard errors in parentheses, * *p* < 0.10, ** *p* < 0.05, *** *p* < 0.01.

**Table 3:** Robust results.

	Dependent Variable: Life Satisfaction				
	(1)	(2)	(3)	(4)	(5)
DID * PM_2.5_	−0.032 **	−0.037 ***	−0.041 **	−0.054 ***	−0.045 ***
	(0.016)	(0.014)	(0.016)	(0.021)	(0.013)
DID	0.129 **	0.134 **	0.173 ***	0.195 **	0.215 ***
	(0.063)	(0.055)	(0.065)	(0.083)	(0.050)
PM_2.5_	−0.049	0.060 **	−0.128 ***	−0.001	0.145 ***
	(0.033)	(0.030)	(0.036)	(0.046)	(0.010)
Controls	Y	Y	Y	Y	Y
Year	Y	Y	Y	Y	Y
Individual	Y	Y	Y	Y	
Constant	4.728 ***	2.787 ***	4.451 ***	2.621 ***	0.433 ***
	(0.724)	(0.410)	(0.502)	(0.944)	(0.006)
*N*	117,232	102,636	98,941	74,239	158,760

Note: Standard errors in parentheses, * *p* < 0.10, ** *p* < 0.05, *** *p* < 0.01.

**Table 4:** IV results.

	2SLS		2SLS		2SLS	
	PM_2.5_	Lifesati	PM_2.5_	Lifesati	PM_2.5_	Lifesati
	(1)	(2)	(3)	(4)	(5)	(6)
DID * PM_2.5_		−0.041 ***		−0.050 ***		−0.041 ***
		(0.012)		(0.012)		(0.012)
DID		0.151 ***		0.201 ***		0.151 ***
		(0.046)		(0.049)		(0.046)
PM_2.5_		−0.068		−0.309 ***		−0.068
		(0.107)		(0.118)		(0.106)
Frequency	0.002 ***					
	(0.000)					
Log(frequency)			0.131 ***			
			(0.002)			
Proportion of total frequency					0.723 ***	
					(0.011)	
Constant	6.251 ***	3.730 ***	6.019 ***	4.648 ***	6.246 ***	3.731 ***
	(0.073)	(0.405)	(0.073)	(0.448)	(0.073)	(0.403)
Controls	Yes	Yes	Yes	Yes	Yes	Yes
Year	Yes	Yes	Yes	Yes	Yes	Yes
Individual	Yes	Yes	Yes	Yes	Yes	Yes
*N*	113,128	152,467	113,128	151,616	113,128	152,467

Note: Standard errors in parentheses, * *p* < 0.10, *** *p* < 0.01.

**Table 5:** Heterogeneity results.

	Dependent Variable: Life Satisfaction									
	Age ≤ 55	Age > 55	Unhealthy	Healthy	Low Edu.	High Edu.	Low Inc.	High Inc.	Rural	Urban
DID * PM_2.5_	−0.038 **	−0.034	−0.032	−0.044 **	−0.031 *	−0.036 **	−0.019	−0.069 ***	−0.012	−0.085 ***
	(0.019)	(0.029)	(0.022)	(0.020)	(0.019)	(0.018)	(0.026)	(0.025)	(0.022)	(0.024)
DID	0.145 *	0.134	0.122	0.153 *	0.104	0.134 *	0.102	0.235 **	0.037	0.329 ***
	(0.075)	(0.114)	(0.088)	(0.079)	(0.074)	(0.070)	(0.103)	(0.099)	(0.088)	(0.097)
PM_2.5_	0.016	−0.120 **	−0.004	0.025	0.021	0.020	−0.082	0.058	−0.104 **	0.047
	(0.040)	(0.059)	(0.046)	(0.044)	(0.043)	(0.037)	(0.055)	(0.052)	(0.051)	(0.048)
Controls	Yes	Yes	Yes	Yes	Yes	Yes	Yes	Yes	Yes	Yes
Year	Yes	Yes	Yes	Yes	Yes	Yes	Yes	Yes	Yes	Yes
Individual	Yes	Yes	Yes	Yes	Yes	Yes	Yes	Yes	Yes	Yes
Constant	4.286 ***	5.514 ***	4.106 ***	2.298 ***	3.280 ***	2.473 ***	7.066 ***	0.982	6.480 ***	1.152
	(0.835)	(1.755)	(0.580)	(0.512)	(0.545)	(0.461)	(1.283)	(1.023)	(1.064)	(1.109)
*N*	78,965	38,267	62,162	68,266	61,891	68,537	56,952	60,280	60,538	56,694

Note: Standard errors in parentheses, * *p* < 0.10, ** *p* < 0.05, *** *p* < 0.01.

**Table 6:** Mechanism analysis—cognitive effect and avoidance effect.

	Environmental Quality Perception			Health Expenditure		
	(1)	(2)	(3)	(4)	(5)	(6)
DID * PM_2.5_	0.231 ***	0.252 ***	0.252 ***	27.288 ***	28.385 ***	28.385 ***
	(0.036)	(0.051)	(0.057)	(4.948)	(4.984)	(7.602)
DID	−0.732 ***	−0.874 ***	−0.874 ***	−131.387 ***	−133.456 ***	−133.456 ***
	(0.140)	(0.202)	(0.221)	(19.237)	(19.551)	(29.819)
PM_2.5_	0.585 ***	0.444 ***	0.444 ***	15.230	13.253	13.253
	(0.089)	(0.112)	(0.121)	(12.290)	(10.326)	(15.545)
Controls		Yes	Yes		Yes	Yes
Year	Yes	Yes	Yes	Yes	Yes	Yes
Individual	Yes	Yes	Yes	Yes	Yes	Yes
Constant	3.557 ***	13.504 ***	13.504 ***	−23.533	−327.851	−327.851
	(0.338)	(2.723)	(2.873)	(46.533)	(202.907)	(217.464)
*N*	123,486	93,314	93,314	124,483	105,669	105,669

Note: Standard errors in parentheses, * *p* < 0.10, *** *p* < 0.01.

**Table 7:** Mechanism analysis—envy effect.

	Low Pollution		Medium Pollution		High Pollution	
	(1)	(2)	(3)	(4)	(5)	(6)
DID * PM_2.5_	0.079 **	0.173 **	−0.212 ***	−0.201 ***	−0.347 ***	−0.333 ***
	(0.032)	(0.081)	(0.052)	(0.055)	(0.116)	(0.126)
DID	−0.121	−0.445 *	0.756 ***	0.718 ***	1.534 ***	1.497 ***
	(0.106)	(0.270)	(0.198)	(0.211)	(0.504)	(0.545)
PM_2.5_	0.116 **	0.041	0.069	0.060	0.014	−0.015
	(0.047)	(0.085)	(0.059)	(0.063)	(0.131)	(0.146)
Controls		Yes		Yes		Yes
Year	Yes	Yes	Yes	Yes	Yes	Yes
Individual	Yes	Yes	Yes	Yes	Yes	Yes
Constant	3.052 ***	1.726	3.187 ***	2.453 ***	3.538 ***	9.709 ***
	(0.146)	(2.483)	(0.227)	(0.614)	(0.572)	(1.616)
*N*	39,349	24,007	77,377	63,620	38,900	31,739

Note: Standard errors in parentheses, * *p* < 0.10, ** *p* < 0.05, *** *p* < 0.01.

## Data Availability

Publicly available datasets were analyzed in this study. This data can be found here: [http://www.isss.pku.edu.cn/cfps/] (accessed on 1 April 2021).

## Appendix A

**Table A1:** Event study results.

	Dependent Variable: Life Satisfaction
Pre6	0.029
	(0.027)
Pre4	−0.034
	(0.023)
Pre2	−0.015
	(0.017)
Post2	−0.062 ***
	(0.022)
Post4	−0.126 ***
	(0.034)
Controls	Y
Year	Y
Individual	Y
Constant	4.555 ***
	(0.782)
*N*	117,086

Note: Standard errors in parentheses, *** *p* < 0.01.
